# Genome-wide association study of preterm birth and gestational age in a Japanese population

**DOI:** 10.1038/s41439-023-00246-9

**Published:** 2023-06-13

**Authors:** Keita Hasegawa, Natsuhiko Kumasaka, Kazuhiko Nakabayashi, Hiromi Kamura, Kayoko Maehara, Yoshifumi Kasuga, Kenichiro Hata, Mamoru Tanaka

**Affiliations:** 1 Department of Maternal-Fetal Biology, National Center for Child Health and Development, Setagaya, Tokyo, 157-8535 Japan; 2 Department of Obstetrics and Gynecology, Keio University School of Medicine, Shinjuku, Tokyo, 160-0016 Japan; 3 Medical Research Center for Japan Environment and Children’s Study, National Center for Child Health and Development, Setagaya, Tokyo, 157-8535 Japan; 4 Department of Human Molecular Genetics, Gunma University Graduate School of Medicine, Maebashi, Gunma 371-8511 Japan; 5 Present Address: Department of Nutrition, Graduate School of Health Sciences, Kio University, Kitakatsuragi, Nara, 635–0832 Japan

**Keywords:** Genome-wide association studies, Clinical genetics

## Abstract

Preterm birth (PTB), defined as the birth of a baby at <37 weeks of gestation, is known to be the main cause of neonatal morbidity and mortality. Here, we report genetic associations between preterm birth and gestational age in a Japanese population. We conducted a genome-wide association study (GWAS) of 384 cases who delivered prematurely and 644 controls and considered gestational age as a quantitative trait in 1028 Japanese women. Unfortunately, we were unable to identify any significant variants associated with PTB or gestational age using the current sample. We also examined genetic associations previously reported in European populations and identified no associations, even with the genome-wide subthreshold (*p* value < 10^–6^). This data report aims to provide summary statistics of current GWASs on PTB in a Japanese population for future meta-analyses of genetics and PTB with larger sample sizes.

## Pregnancy: Searching for genes for preterm birth

The genes associated with preterm birth, defined as birth before 37 weeks of gestation and a main cause of infant mortality, have yet to be identified in the Japanese population. Such genes have been identified in European populations. Natsuhiko Kumasaka at the National Center for Child Health and Development in Tokyo, Japan, and co-workers conducted a genome-wide association study of preterm birth or weeks of gestation using blood samples from over a thousand women. However, they found no significant associations, even when they examined regions centered on genes identified in previous studies. Identifying associated genes could help to identify and reduce risks to infants. The authors note that their data could be included in meta-analyses that combine the results of many studies and recommend further studies with larger sample sizes.

## Introduction

Preterm birth (PTB) is defined as delivery at <37 weeks of gestation. The estimated global PTB rate was 9.8% in 2000 and increased to 10.6% in 2014, ranging from 13.4% in North Africa to 8.7% in Europe^1^. The percentage of PTB in Japan was 3.71% between 1979 and 1983 but increased to 4.77% between 2009 and 2014^2^. Although the rate of PTB in Japan is lower than that worldwide, it is clear that PTB is closely associated with morbidity and mortality in immature infants, and elucidating the cause of PTB is of interest. Many maternal and fetal characteristics have been associated with PTB, including maternal demographic characteristics, nutritional status, pregnancy history, infection, and biological and genetic markers^3^. However, 31–50% of PTB are due to spontaneous onset of labor, making it difficult to identify the cause^4^. The association between PTB and genetic variants has been reported previously. It was reported that women whose mothers or sisters had a history of PTB were 55% more likely to deliver prematurely than those with no family history of PTB^5^, and that genetic factors accounted for 34% of delivery timing in twin analysis^6^. Moreover, it was reported that maternal and fetal genetic factors influenced gestational age^7^. Recently, several genome-wide association studies (GWASs) on PTB have been conducted. The largest study, in which the EBF1, EEFSEC, AGTR2, WNT4, ADCY5, and RAP2G loci were associated with gestational week, and the EBF1, EEFSEC, and AGTR2 loci were associated with PTB, was reported in 2017 and used a discovery dataset from 23andMe comprising over 40 thousand women of European ancestry^8^. In addition, a genome-wide association study (GWAS) of the Finnish population in 2019 reported a novel association between the SLIT2 locus and PTB^9^. Although a few GWASs on PTB and gestational age have been reported, no GWASs have been conducted in an Asian population, including the Japanese population. Therefore, in this study, we conducted a GWAS of PTB and gestational age to identify related variants in a Japanese population.

## Materials and methods

We collected 1158 samples of postpartum maternal blood from Keio University Hospital between 2011 and 2020. This study was approved by the Keio University School of Medicine Ethics Committee (20100154) and the Institutional Review Board of the National Research Institute for Child Health and Development (406) and was conducted in accordance with the ethical standards outlined in the 1964 Helsinki Declaration and later amendments. All women provided written informed consent.

Of the 1158 samples, 80 medically-indicated PTB samples, such as those from women with multiple pregnancies and placenta previa, were excluded. After excluding these 80 samples, analysis was performed, as shown in the flowchart (Supplementary Fig. 1). Genome-wide SNP genotyping was performed using the Infinium Omni2.5-8 v1.0 Kit and v1.1 Kit (Illumina, San Diego, CA, USA) by using the standard cluster file provided by Illumina. The ped file generated using GenomeStudio v2.0.5 (Illumina, San Diego, CA, USA) was converted to the Variant Call Format (VCF) file using PLINK 1.90 beta^10^. The VCF file was converted from Illumina strands to reference sequence strands (GRCh37) using bcftools 1.9^11^. The VCF files were converted from GRCh37 to GRCh38 using CrossMap 0.6.0 (default: no option)^12^.

By using the converted VCF files, we filtered out low-quality samples by applying the following conditions. For sample call rates, the PLINK command with the “--missing” option was used, and no samples were removed because all samples had call rates above 0.98. For heterozygosity rates, the PLINK command with the “--het” option was used, and nine samples were removed because their heterozygosity rates were outliers ±3 SD from the 1078 samples. For sex inconsistency, we calculated the heterozygosity rate of each sample on chromosome X, and four samples were removed because their heterozygosity rates were less than 0.01.

After removing 13 samples by sample QC, we also filtered out low-quality variants by applying the following conditions. For variant call rates, the PLINK command with the “--missing” option was used, and 42,469 variants in the VCF files of ver1.0 and 65,843 variants in the VCF files of ver1.1 were removed because these variants had call rates less than 0.98. For Hardy–Weinberg equilibrium (HWE), the PLINK command with the “--hardy” option was used, and 408 variants in the VCF files of ver1.0 and 624 variants in those of ver1.1 were removed (*p* value of HWE <1e−6).

After removing low-quality variants by variant QC, the resultant VCF files of ver1.0 and ver1.1 were transformed into a form that conforms to Beagle 5.4 (default: no option)^13^ and integrated with shared SNPs before imputation. By using this conformed file, whole genome imputation was performed with the 1000 Genomes Project reference haplotype data (http://ftp.1000genomes.ebi.ac.uk/vol1/ftp/data_collections/1000G_2504_high_coverage/working/20201028_3202_phased/). Beagle software was used to estimate the genotype dosages for the GWASs, and the variants with DR2 > 0.7 were retained. We also filtered out low-quality imputed variants by applying the Hardy–Weinberg equilibrium (HWE) test with a *p* > 0.001 and minor allele frequency (MAF) > 0.001.

After removing low-quality variants by variant QC, we checked the genetic relatedness. For the detection of relatedness, the PLINK command with the “--genome --min 0.1875” option was used, and 37 pairs were indicated to show relatedness. Of these 37 pairs, 7 pairs (14 samples) were from the same person who delivered twice: one delivery was a term birth and the other was a preterm birth. Of these pairs, 7 samples from the preterm birth were used for the subsequent analysis. Twenty-one pairs (42 samples) were from the same person who delivered twice, and both were term births. In these pairs, 21 samples with higher sample call rates were used for the subsequent analysis. The remaining 9 pairs (18 samples) were not from the same person. In those pairs, 9 samples with higher sample call rates than the other samples were used for the subsequent analysis. In summary, by analyzing relatedness, 37 samples were unrelated and used for the subsequent analysis. After filtering based on genetic relatedness, a total of 1028 samples and 11,183,581 variants were analyzed.

By using the resultant file, detection of population stratification was conducted by using principal component analysis (PCA) (Supplementary Fig. 2). Thirteen samples were identified as Han Chinese samples, but they were not removed because the top 10 genotype principal components (PCs) were used as covariates in the subsequent analysis.

GWASs were independently performed for both preterm birth as a binary trait and gestational age as a continuous trait by using PLINK 1.90 beta. In the analysis of preterm birth, the phenotype data were divided into two groups: the term-birth group (control group; *N* = 644) and the preterm-birth group (case group; *N* = 384) (Supplementary Fig. 3a). Of 1028 samples, 578 were derived from the VCF files of ver1.0, and 450 samples were derived from the VCF files of ver1.1 (Supplementary Fig. 3b). In the analysis of gestational age, because of the variability of the gestational age in the sample, quantile normalization was performed; the values were considered quantitative traits in the GWASs (Supplementary Fig. 3c). In both analyses, the version of Illumina cluster files (1.0 and 1.1) and the first 10 principal components (generated as a result of PCA using samples from the Japanese population in Tokyo and the Han Chinese population extracted from the 1000 Genome Project data) were used as covariates. We set the genome-wide significance level at *p* < 5.0 × 10^–8^.

## Results

**Fig. 1:**
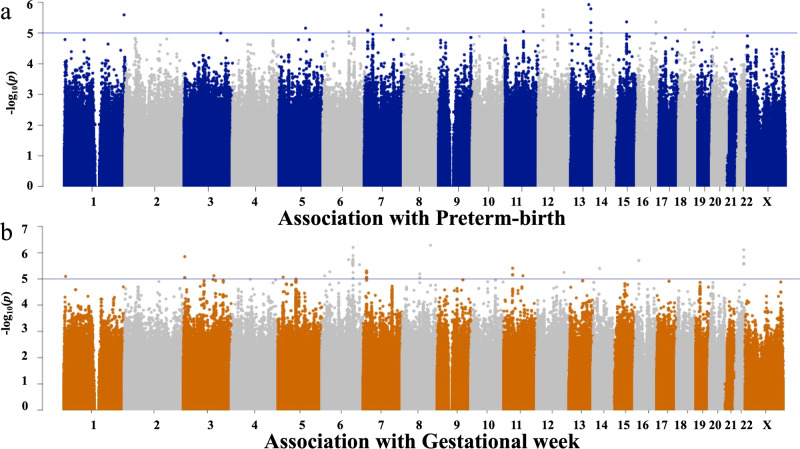
Manhattan plots of the GWAS data. Each dot represents a variant. The *x*-axis represents genomic coordinates, and the *y*-axis represents −log10 (*p* value). Two colors, blue and gray, are used alternately for dots on a chromosome for easy viewing. The blue line represents the suggestive significant threshold (*α*) of *p* = 1.0 × 10^−5^. **a** Manhattan plot of genome-wide associations between variants and PTB among the Japanese population (384 cases and 644 controls). **b** Manhattan plot of genome-wide associations between variants and gestational age among the Japanese population.

A GWAS of PTB was conducted, and no SNPs were identified as significant at *p* < 5.0 × 10^–8^ (Fig. 1a). Then, a GWAS of gestational age (as a quantitative trait) was conducted, and no SNPs were identified as significant at *p* < 5.0 × 10^–8^ (Fig. 1b). Thus, in this study, no significant SNPs were found to be associated with PTB or gestational age with the current sample (Supplementary Fig. 4).

**Fig. 2:**
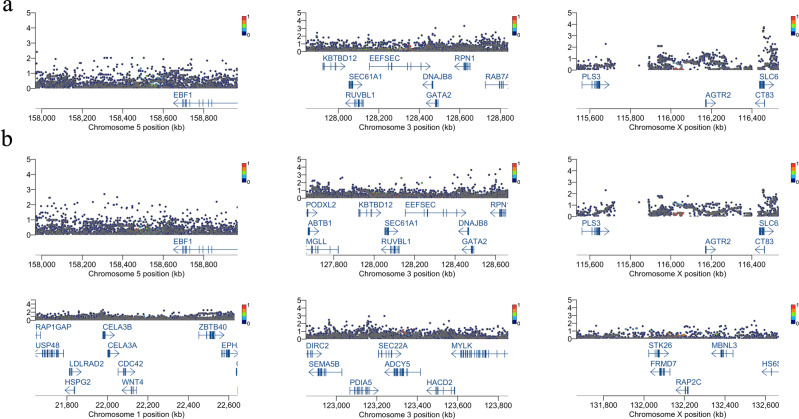
Regional plots of the GWAS results within the ±500 kb interval of the SNP loci known to be associated with PTB and gestational age. The *x*-axis represents the chromosomal position. The *y*-axis represents −log10 (*p* value). Each dot represents a variant, and its color shows the LD index (*r*^2^) with the known associated SNP in each locus. Gene names and nucleotide coordinates are shown in each panel. **a** Regional plots for associations between variants and PTB among the Japanese population (384 cases and 644 controls). **b** Regional plots for associations between variants and gestational age among the Japanese population.

The regions centered on SNPs known to be associated with PTB and gestational age were examined (Fig. 2 and Supplementary Table 1). None of the known SNPs were associated with PTB (Fig. 2a) or gestational age (Fig. 2b), even with a genome-wide subthreshold of *p* < 10^–6^. Odds ratios of known SNPs^8^ associated with PTB and effect sizes of SNPs^8^ associated with gestational age were compared to those from our study (Supplementary Fig. 5).

## Discussion

We conducted a GWAS in a Japanese population and were unable to identify any significant variants associated with PTB and gestational age. We also examined variants around the variants known to be associated with PTB and gestational age reported previously, but no significant variants were confirmed. There are a couple of reasons that could explain these negative findings in this study. Although we excluded samples from medically-indicated PTBs in this study, we did not completely focus on spontaneous PTB because the sample size was expected to be extremely small. The sample size of this study was much smaller than that of other studies. Further studies with larger sample sizes and detailed clinical information are indispensable to detect significant genetic variants associated with PTB and gestational age in the Japanese population.

### Supplementary information

Supplementary Figure 1

Supplementary Figure 2

Supplementary Figure 3

Supplementary Figure 4

Supplementary Figure 5

Supplementary Table 1

Supplementary Figure Legends

## Data Availability

The summary statistics of GWASs on PTB and gestational weeks will be deposited in the GWAS Catalog (Accession Numbers: GCST90268004 and GCST90268005).
