# Supplementary figures and images for: Genome-wide association study of preterm birth and gestational age in a Japanese population
Source: Hum Genome Var. 2023 Jun 13;10:19. doi: 10.1038/s41439-023-00246-9 (PMC10264385; doi:10.1038/s41439-023-00246-9)

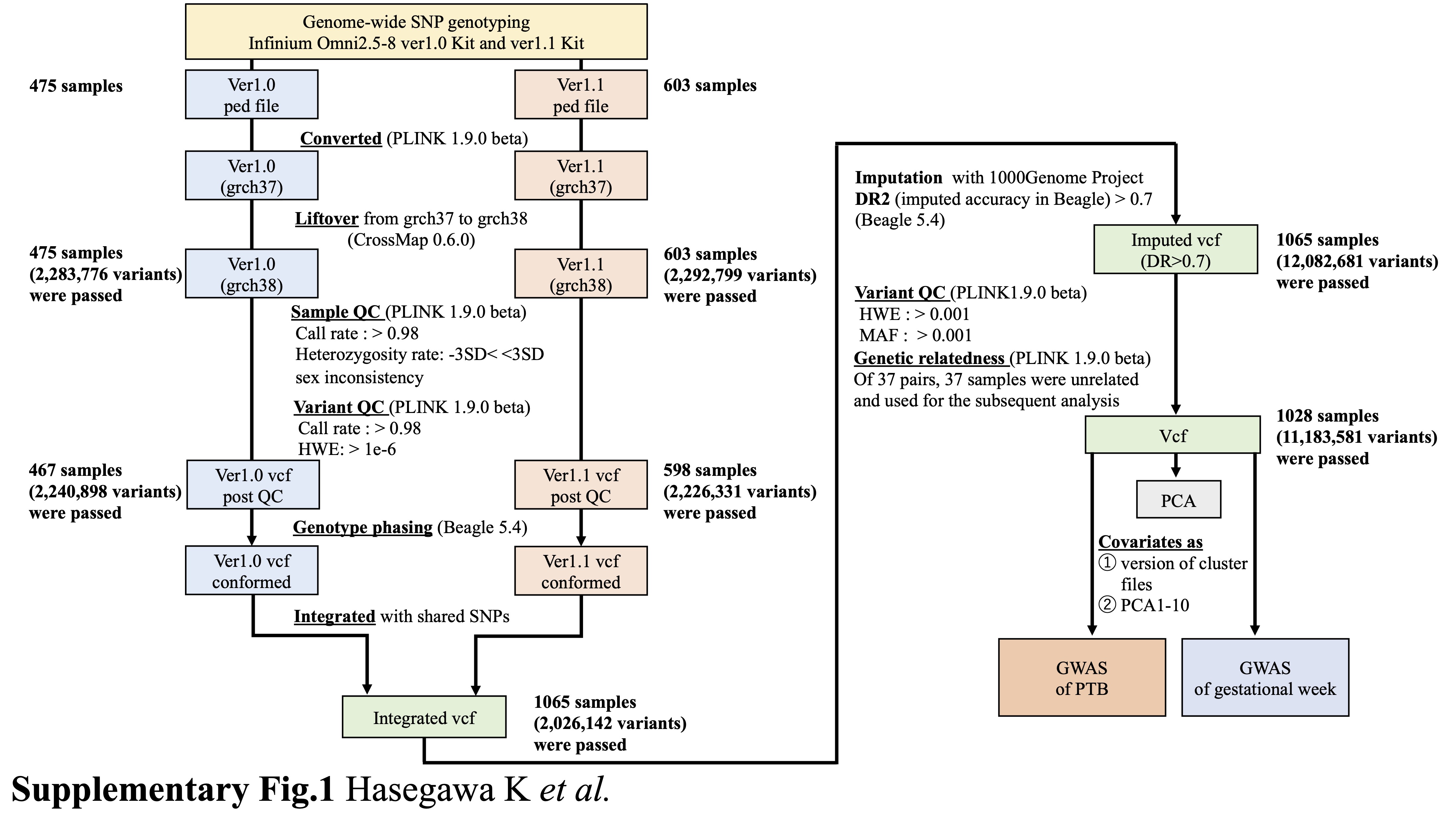

Supplement: Supplementary file 1 — Supplementary Figure 1 [file 41439_2023_246_MOESM1_ESM.jpg]

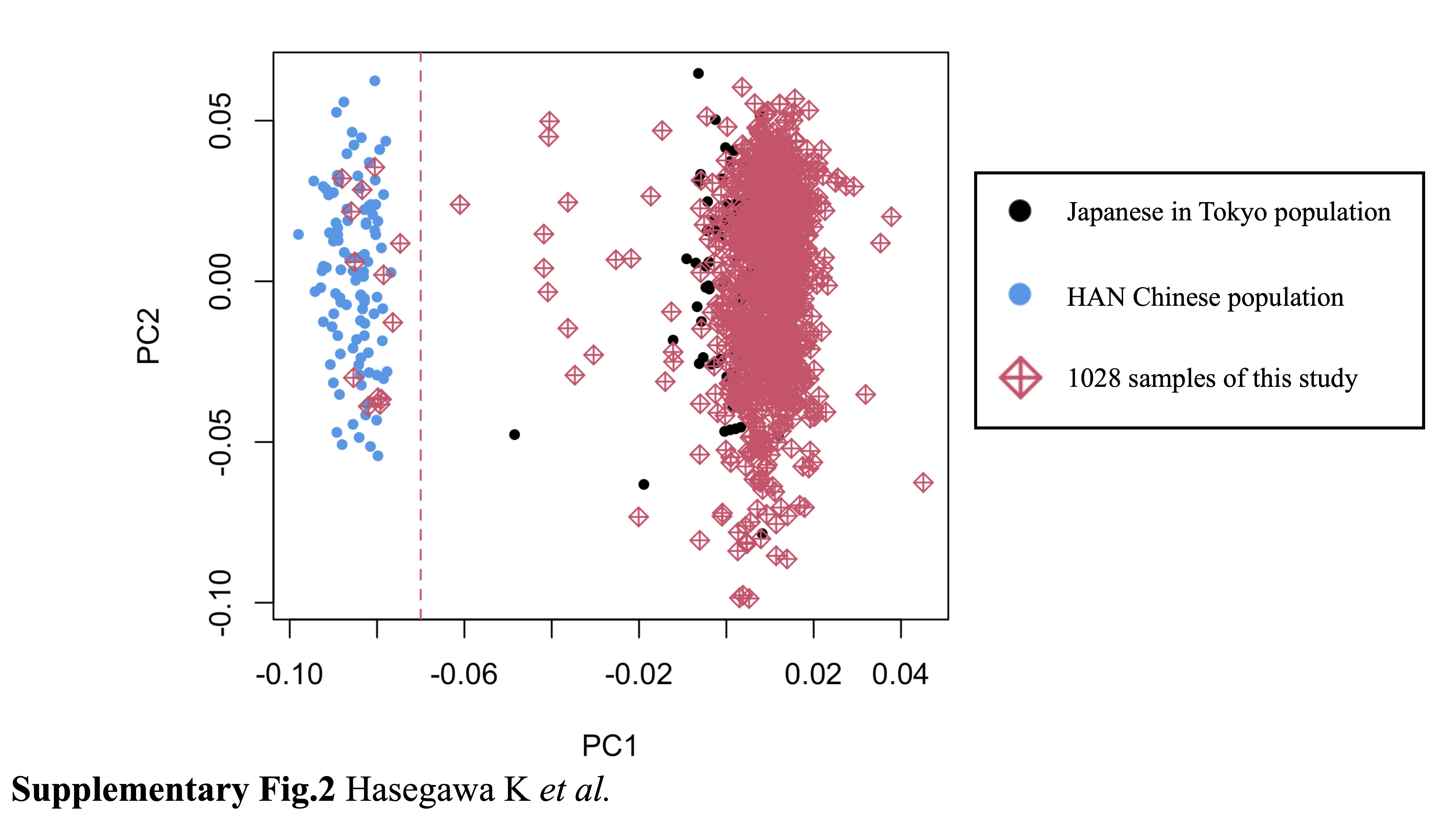

Supplement: Supplementary file 2 — Supplementary Figure 2 [file 41439_2023_246_MOESM2_ESM.jpg]

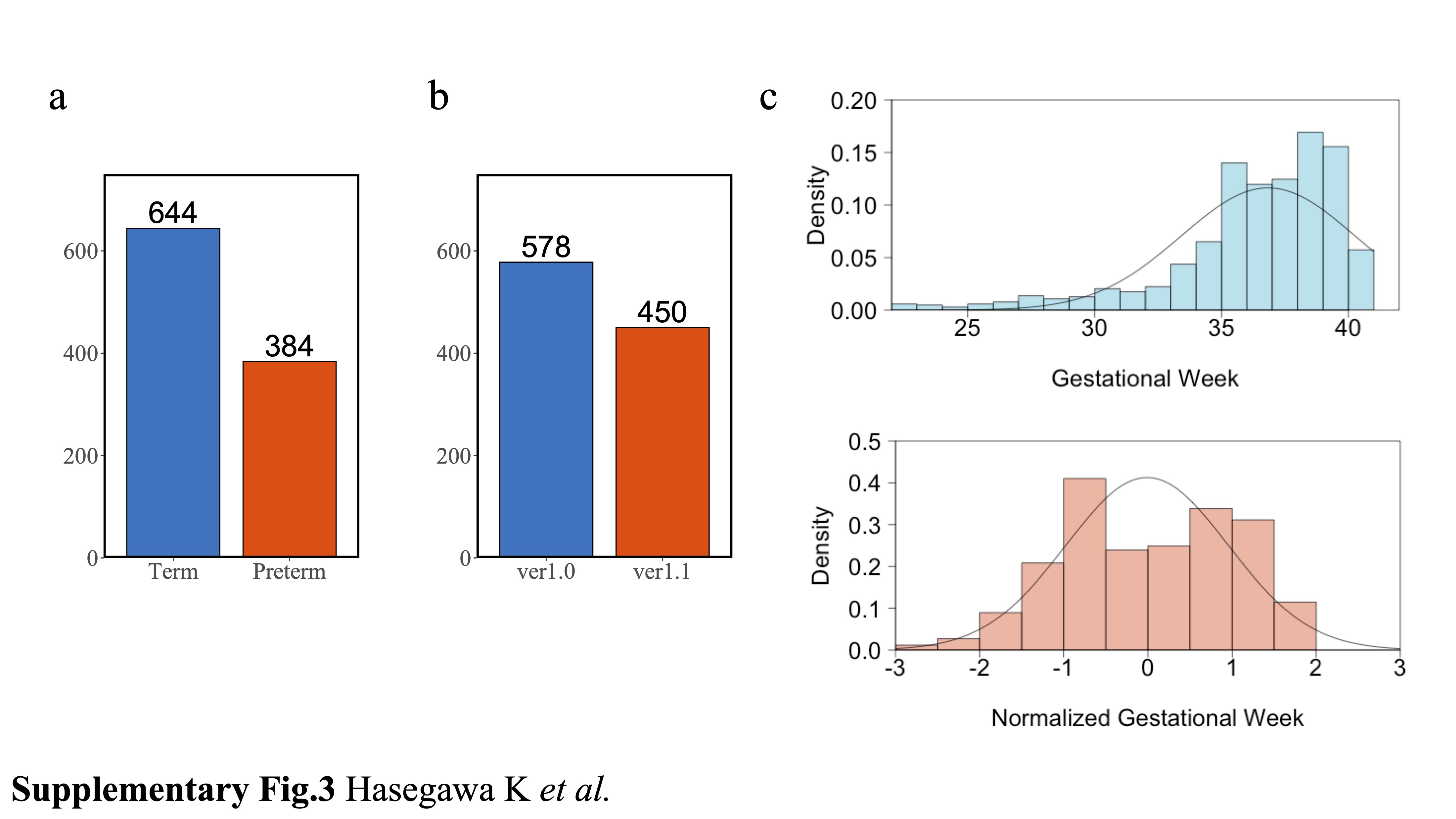

Supplement: Supplementary file 3 — Supplementary Figure 3 [file 41439_2023_246_MOESM3_ESM.jpg]

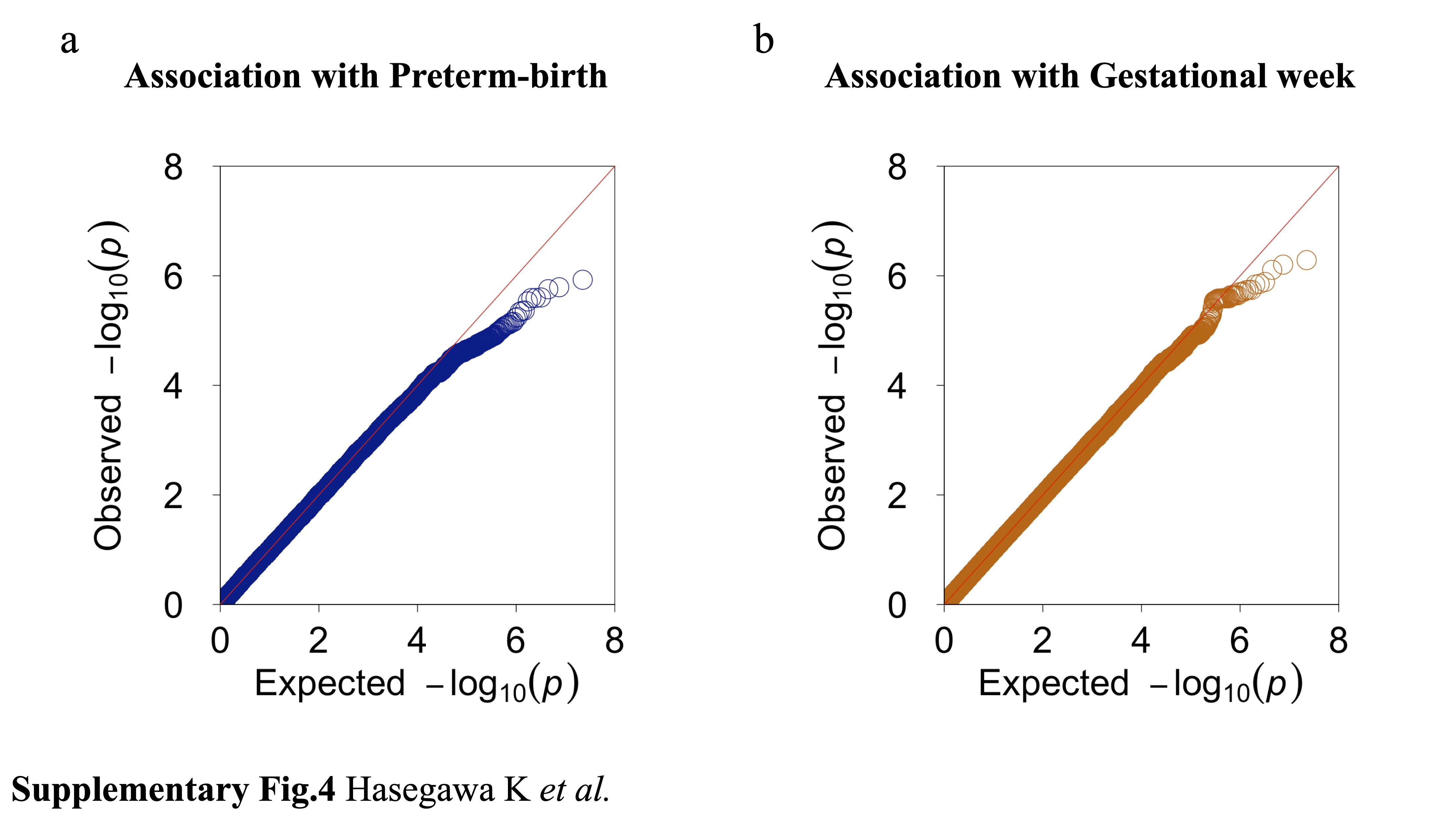

Supplement: Supplementary file 4 — Supplementary Figure 4 [file 41439_2023_246_MOESM4_ESM.jpg]

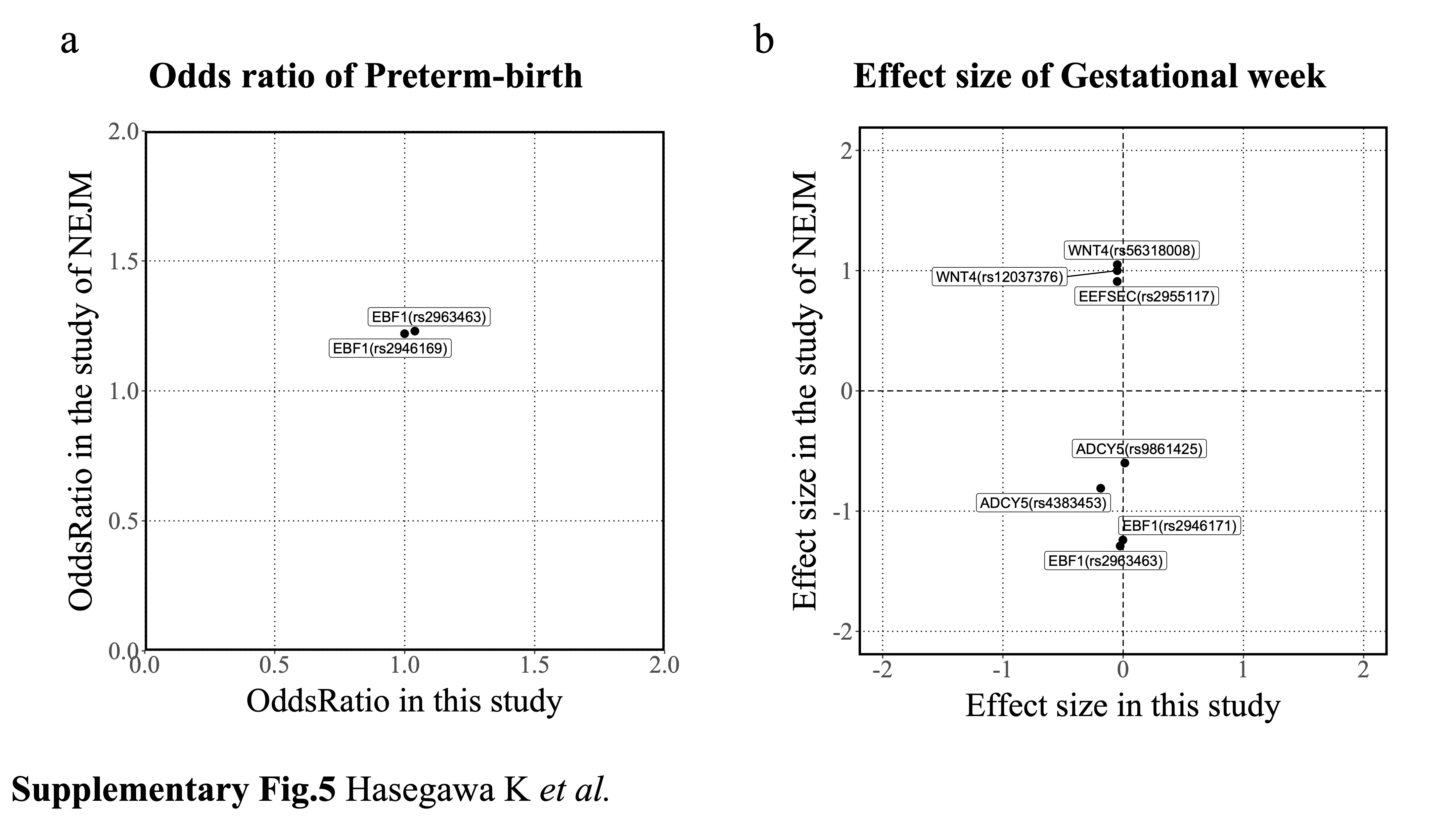

Supplement: Supplementary file 5 — Supplementary Figure 5 [file 41439_2023_246_MOESM5_ESM.jpg]

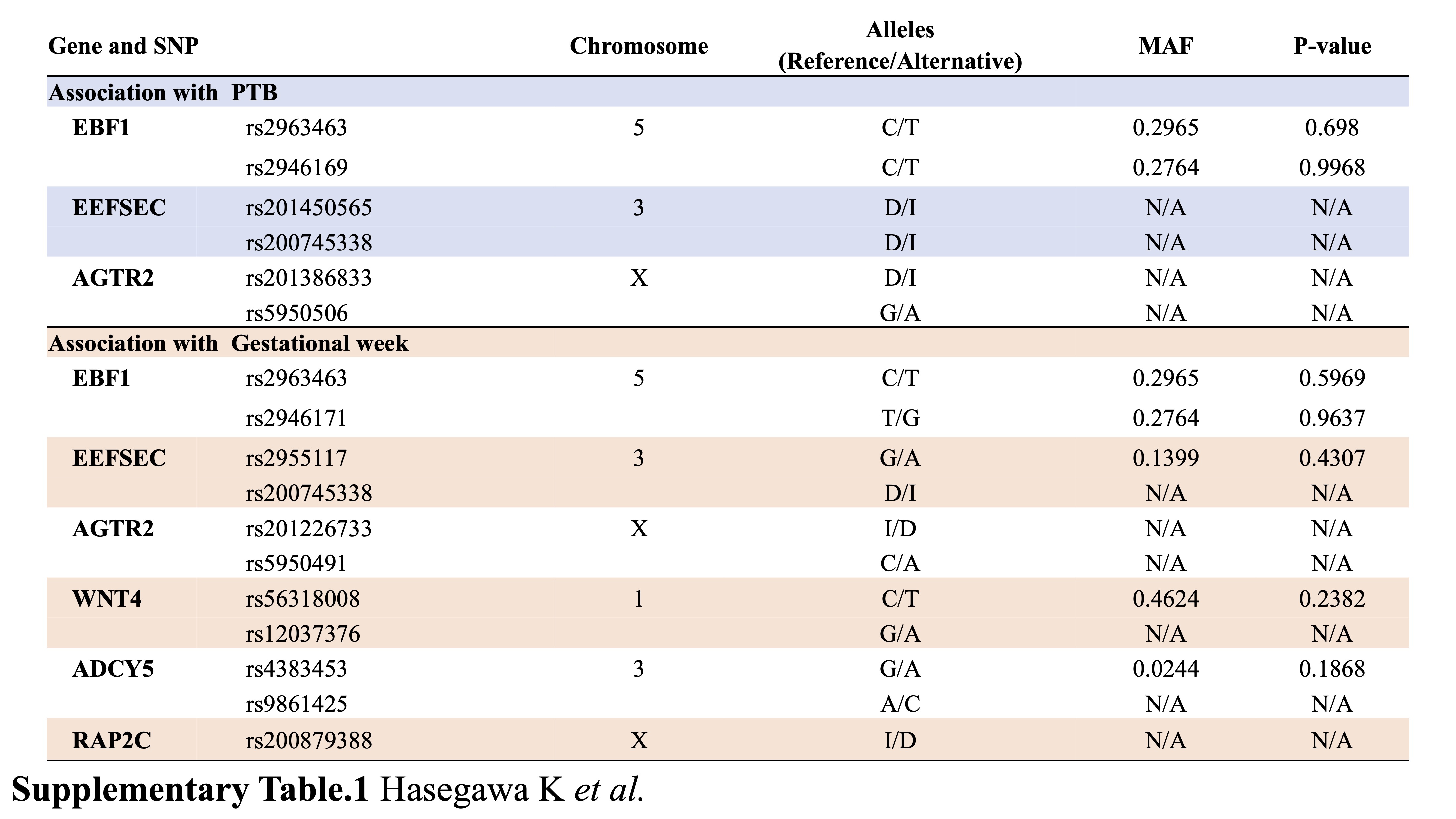

Supplement: Supplementary file 6 — Supplementary Table 1 [file 41439_2023_246_MOESM6_ESM.jpg]
