# Supplementary material for: Genome-wide association study of preterm birth and gestational age in a Japanese population
Source: Hum Genome Var. 2023 Jun 13;10:19. doi: 10.1038/s41439-023-00246-9 (PMC10264385; doi:10.1038/s41439-023-00246-9)
Supplement: Supplementary file 7 — Supplementary Figure Legends [file 41439_2023_246_MOESM7_ESM.docx]

**Supplementary Figure 1.** Flowchart of the analysis in this study.

**Supplementary Figure 2.**

Principal component analysis (PCA) plot for estimating population structure and sample ancestry in genetic datasets. The x-axis represents principal component 1 (PC1). The y-axis represents principal component 2 (PC2). Each dot represents an individual sample. The blue dot represents the Han Chinese population, and the black dot represents the Japanese population in Tokyo in the 1000 Genomes Project reference haplotype data. The red diamonds represent the samples in this study. The red dotted line represents the cutoff value of -0.07 in PC1.

**Supplementary Figure 3.** Characteristics of the participants in this study.

**a.** The number of individuals with term births (controls) and preterm births (cases).

**b.** The number of samples analyzed by the Infinium Omni2.5-8 Kit versions 1.0 and 1.1.

**c.** Histograms of gestational age: raw data (upper) and quantile-normalized data (lower).

**Supplementary Figure 4.** Quantile‒quantile (QQ) plots of observed versus expected P values of the GWAS results. The x-axis represents the expected −log10 (p value). The y-axis represents the observed −log10 (p value). The red line is the line of Y=X.

**a.** QQ plot of GWAS data based on preterm birth data.

**b.** QQ plot of GWAS data based on gestational age data.

**Supplementary Figure 5.** Scatter plots of the odds ratios of known SNPs^8^ associated with PTB and the effect sizes of SNPs with gestational age. The corresponding gene name and rsID numbers are labeled near each dot.

**a.** Scatter plot of the odds ratios of SNPs associated with preterm birth. The x-axis represents the odds ratios in this study. The y-axis represents the odds ratios of the previous report^8^.

**b.** Scatter plot of the effect sizes of SNPs associated with gestational age. The x-axis represents the effect sizes in this study. The y-axis represents the effect sizes of the previous report^8^.

**Supplementary Table 1.** Characteristics of the SNPs known to be associated with preterm birth and gestational age.
